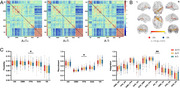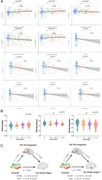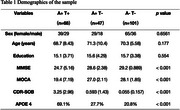# Decreased Audio‐Visual Network Integration Mediates Amyloid‐related Tau Spreading

**DOI:** 10.1002/alz70856_105624

**Published:** 2026-01-09

**Authors:** Jieying Li, Lin Gan, Gleb Bezgin, Tevy Chan, Brandon J Hall, Nesrine Rahmouni, Yi‐Ting Wang, Etienne Aumont, Seyyed Ali Hosseini, Kely Monica Quispialaya Socualaya, Lydia Trudel, Joseph Therriault, Arthur C. Macedo, Jaime Fernandez Arias, Yansheng Zheng, Delphine Olivia‐Lopez, Maxime Montembeault, Rong Li, Pedro Rosa‐Neto

**Affiliations:** ^1^ Translational Neuroimaging Laboratory, The McGill University Research Centre for Studies in Aging, Montreal, QC, Canada; ^2^ Sichuan Provincial People's Hospital, School of Medicine, University of Electronic Science and Technology of China, Chengdu, QC, Canada; ^3^ University of Electronic Science and Technology of China, Chengdu, Sichuan, China; ^4^ Translational Neuroimaging Laboratory, The McGill University Research Centre for Studies in Aging, Montréal, QC, Canada; ^5^ McGill University, Montreal, QC, Canada; ^6^ Neuroinformatics for Personalized Medicine lab, Montreal Neurological Institute, McGill University, Montreal, QC, Canada; ^7^ Montreal Neurological Institute, Montreal, QC, Canada; ^8^ Université du Québec à Montréal, Montréal, QC, Canada; ^9^ Translational Neuroimaging Laboratory, McGill Research Centre for Studies in Aging, Montreal, QC, Canada; ^10^ University of Pavia, Pavia, Pavia, Italy; ^11^ Douglas Mental Health University Institute, Montréal, QC, Canada

## Abstract

**Background:**

Growing evidence indicates that the coexistence of visual and auditory impairments increases the risk of developing Alzheimer's disease (AD). However, the mechanisms through which these sensory deficits influence the progression of AD, particularly their impact on amyloid and tau pathology, remain unclear. We hypothesize that alterations in the audio‐visual dynamic network play a critical role in mediating the spread of amyloid‐related tau pathology during the early stages of AD.

**Method:**

This study included multimodal imaging data, including functional MRI, [^18^F]NAV4694 amyloid‐PET, and [^18^F]NAV4694 tau‐PET, from the TRIAD cohort (*n* = 216, Table 1). Participants were classified as amyloid‐beta (Aβ) positive (A+) or negative (A−) based on established global uptake values of [^18^F]NAV4694 (global standardized uptake value ratio [SUVR] > 1.55). Tau positivity (T+) or negativity (T−) was determined using [^18^F]MK6240, with a temporal meta‐ROI SUVR threshold > 1.30. Tau staging was based upon Braak stage classification. Brain dynamics in resting‐state fMRI data were analyzed with a multilayer modularity algorithm in MATLAB, focusing on primary sensory and higher‐order networks.

**Result:**

Module allegiance within the auditory network (AN) and visual networks (VN) was lower in the A+T+ group compared to the A−T− group. Additionally, flexibility within the frontoparietal network (FPN) was increased, while recruitment within the FPN and integration between AN and VN were reduced in the A+T+ group compared to A−T− group (Figure 1). Integration between AN and VN negatively correlated with [^18^F]MK6240 SUVR in Braak stage 1 through 5 and the temporal meta‐ROI, as well as with neocortical [^18^F]NAV4694 SUVR. Furthermore, AN‐VN integration mediated the relationship between neocortical [^18^F]NAV4694 SUVR and [^18^F]MK6240 SUVR in Braak stage 1 and 2 (Figure 2).

**Conclusion:**

Our study suggests that audio‐visual network integration during the early stages of tau pathology mediates amyloid‐related tau accumulation. This supports a framework in which decline brain network integration may facilitates the early spread of amyloid‐driven tau pathology across interconnected brain regions.